# CRISPR Screen Contributes to Novel Target Discovery in Prostate Cancer

**DOI:** 10.3390/ijms222312777

**Published:** 2021-11-26

**Authors:** Takuya Tsujino, Kazumasa Komura, Teruo Inamoto, Haruhito Azuma

**Affiliations:** 1 Department of Urology, Osaka Medical and Pharmaceutical University, Osaka 569-8686, Japan; teruo.inamoto@ompu.ac.jp (T.I.); haruhito.azuma@ompu.ac.jp (H.A.); 2 Division of Urology, Department of Surgery, Brigham and Women’s Hospital, Harvard Medical School, Boston, MA 02115, USA; 3 Translational Research Program, Osaka Medical and Pharmaceutical University, Osaka 569-8686, Japan

**Keywords:** prostate cancer, CRISPR screen, CRISPR/Cas9

## Abstract

Prostate cancer (PCa) is one of the common malignancies in male adults. Recent advances in omics technology, especially in next-generation sequencing, have increased the opportunity to identify genes that correlate with cancer diseases, including PCa. In addition, a genetic screen based on CRISPR/Cas9 technology has elucidated the mechanisms of cancer progression and drug resistance, which in turn has enabled the discovery of new targets as potential genes for new therapeutic targets. In the era of precision medicine, such knowledge is crucial for clinicians in their decision-making regarding patient treatment. In this review, we focus on how CRISPR screen for PCa performed to date has contributed to the identification of biologically critical and clinically relevant target genes.

## 1. Introduction

Prostate cancer (PCa) accounts for 1.6 million cases and 366,000 deaths worldwide each year [1]. Within the USA, PCa is a common cancer and the second leading cause of cancer-related deaths among men [2]. Androgens are critical factors that promote the growth of PCa cells, and androgen-deprivation therapy (ADT) is the mainstay of treatment for men with metastatic PCa [3]. While initially effective, patients with metastatic PCa receiving ADT will eventually develop resistance and progress to inevitably lethal metastatic castration-resistant prostate cancer (mCRPC) [4]. The last decade has seen an expansion of drugs to prolong the life of mCRPC, including second-generation androgen receptor (AR) inhibitors, the chemotherapeutic taxane cabazitaxel and PARP inhibitors for tumors with defects in DNA-damage-repair proteins such as BRCA1/2 and ATM [5,6,7,8,9,10,11]. However, identifying new therapeutic targets for mCRPC patients and providing new genetic markers for existing therapies remain a critical challenge.

Recent advances in genome editing technology using the clustered regularly interspaced short palindromic repeats/Cas9 (CRISPR/Cas9) [12] have provided a robust and unbiased tool for conducting genetic screens to study biological systems in a genome-wide manner, which is ideal for the identification of target genes. Prior to the CRISPR/Cas9, the functional genetic screen employed RNA interference (RNAi) oligonucleotides for research on loss-of-function and cDNA overexpression libraries for research on the gain of function [13,14,15,16]. However, the construction of cDNA overexpression libraries is challenging. Furthermore, a side-by-side comparison with RNAi knockdown analysis revealed the advantages of functional genomic KO screen using CRISPR/Cas9 [17]. Accordingly, a number of wild-type (WT) Cas9-based CRISPR-KO screens have been performed to date. In more recent years, an increasing number of studies have also utilized mutants of catalytically dead Cas9 (dCas9), in which the nuclease of WT Cas9 is mutated to render it non-functional [18,19]. dCas9 has been fused to a range of chromatin modifier fusion proteins to convert it into a highly versatile enzyme that can be used to perform CRISPR activation (CRISPRa) or repressive CRISPR interference (CRISPRi) screens [19,20]. In this review, the basic concepts underlying different types of screening approaches, including methodologies, will be discussed in turn, followed by studies conducted to date employing such screen strategies to identify target genes in PCa. Throughout, we will discuss the importance of understanding the molecular features of castration-resistant prostate cancer (CRPC) and identifying targets through genomic studies, such as CRISPR screens, in performing precision medicine and conducting clinical trials.

## 2. Methodology of CRISPR Screen

**Figure 1:**
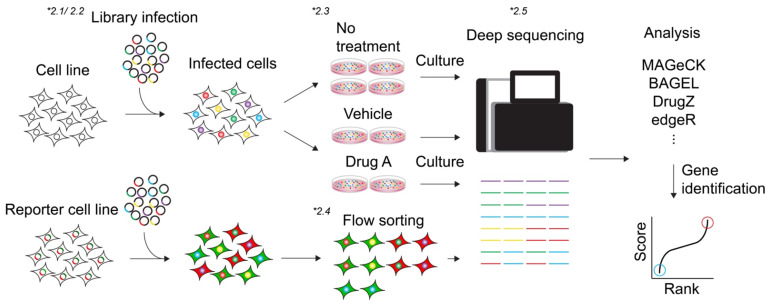
Schematic overview of CRISPR screen. The sgRNA library is transduced into the cells of interest, followed by phenotype selection. For the viability-based screens, infected cells are either cultured as usual or subjected to the selective stress (typically specific drug, etc.) to achieve the phenotype. For the marker selection, cells are sorted by FACS. Followed by the selection, genomic DNA is harvested and the encoded sgRNA is amplified by PCR and identified by NGS. Candidate hits are determined by computational algorithms, which provide the scores as the value of each sgRNA. Hits are ranked by their relative enrichment scores of the sgRNAs (e.g., day0 vs. day28, or non-selected control vs. selected sample). * indicate the corresponding section number in each step.

The schematic overview is shown in Figure 1.

### 2.1. Library

Several pooled libraries for KO screen are available from browsing source Addgene, e.g., GeCKO, H1/H2, Brunello and TKO CRISPR-KO libraries [21,22,23,24]. These libraries contain over 18,000 genes with 4–10 single guide RNAs (sgRNA) per gene. Similarly, CRISPRa and SAM libraries for activation screen and CRISPRi libraries for repression screen are also shared [25,26,27]. Custom libraries are useful for specific studies of interest [28].

### 2.2. Viral Packaging of Library and Transduction

The first step in pooled screen using the CRISPR/Cas9 system is to generate a library of perturbed cells with lentiviral infection of an sgRNA library. Viruses are produced by transfecting an sgRNA library into appropriate host cells, e.g., HEK 293FT cells with superior virus production capacity. To avoid confusion in interpretation in case the host cells take up multiple sgRNAs and target multiple genes per cell, low (~0.3) multiplicity of infection (MOI) is ensured by empirically determining the viral titer [27,29,30]. It is important to note the limitation in this step—some models, such as NCI-H660 and VCAP cells of PCa, make it quite challenging to implement tools through lentiviral approaches.

### 2.3. Viability-Based Screens

One of the most basic experiments to conduct is to identify genes that impact cell fitness. Since perturbations that decrease the cell fitness will be either depleted or completely absent by the end of the screen, this type of screen is termed a negative selection screen. Negative selection screens are most commonly performed in the field of cancer biology to identify dependencies of tumor cells due to specific mutations, copy number alterations, expression patterns and other targets [24,31,32,33,34]. One of the simplest forms of negative selection screen is to continuously culture cells for extended periods of time in order to identify genes required for cell growth. Such screens have been used to identify both the essential genes required for the cell line tested and a small set of genes that are gene-dependent in a particular cancer cell line [35,36,37]. Another negative selection screen form, performed in cell lines with a given genetic background, is the basis for identifying synthetic lethal interactions, in which simultaneous inhibition of two genes impairs cell viability [38]. The discovery of synthetic interactions will enable targeted therapy of cancer cells, making drugs work only on cells with specific alterations, and can offer a new approach to cancer treatment.

The alternative to the negative selection screen is the positive selection screen, which focuses on cells that have been enriched in the course of time. These screens have been used to identify perturbations which confer resistance to small molecules [14,17,39], conditions [40] and pathogen infections [41,42,43,44,45]. In a positive selection screen, most of the population is eliminated, and the few surviving perturbations may become over 100-fold enriched. Eventually, a single screen can result in both positively and negatively selected phenotypes. For example, a viability-based KO screen of a cancer cell line can reveal both oncogene depletion and enrichment of tumor suppressors. Typically, an intermediate dose of a small molecule can identify both sensitization genes and resistance genes [46].

### 2.4. Marker Selection Screen

The marker selection screen aims to identify genetic elements which affect the expression of a particular reporter molecule, and the phenotype is not based on cell viability but on mutations that impact the expression of the marker protein. In this type of screen, the reporter can be genetically engineered by replacing the coding sequence of the gene of interest with the fluorescent marker. Ultimately, fluorescence-activated cell sorting (FACS) allows for the identification of upstream expression regulators by sorting cells with sgRNAs targeting genes which affect the expression of the marker [27,47].

### 2.5. Analysis (Algorithms)

Following the selection step, DNA is collected from surviving cells or FACS-selected cells, and PCR is employed to isolate genomic DNA from the cell population and read the genes responsible for the phenotype. Subsequently, large-scale parallel sequencing is performed using next-generation sequencing (NGS) to cover the regions coding for sgRNAs. Several existing algorithms such as Model-based Analysis of Genome-wide CRISPR/Cas9 KO (MAGeCK) [48,49,50], edgeR [51], Bayesian Analysis of Gene EssentiaLity (BAGEL) [52], CRISPR AnalyzeR for Pooled Screens (caRpools) [53], Platform-independent Analysis of Pooled Screens using Python (PinAPL-Py) [54] and DrugZ [55] can then be used to determine the candidate genes responsible for the observed phenotypes through examining the differences in sgRNA abundance between control and phenotypic samples.

### 2.6. Validation

Analysis of the screen provides a ranked list of candidate genes causing the phenotype. In order to assess which genes contribute to the phenotype and how much, validation is essential. The most critical validation method is to assess whether the selected phenotype is indeed reproducible by introducing sgRNAs targeting the gene of interest. Recent improvements in the specificity of sgRNAs have relieved the need to confirm binding to the target, as long as multiple sgRNAs directed to the same genetic element elicit the phenotype. However, if required, analyses such as genomic PCR, RT-qPCR and Western blotting can be performed to evaluate the functional modification of the targeted gene [25,27]. It is important to note that the confirmation of on-target activity does not exclude the possibility of phenotyping due to off-target effects. Hence, continuous validation experiments of phenotypes using not only one sgRNA with the highest on-target activity but also multiple sgRNAs for each gene are critical. Rescue experiments are another way to confirm whether the genetic entity confers the phenotype. The goal is to confirm whether restoring candidate expression to physiological levels in CRISPR/Cas9-edited cells returns the cells to their wild-type state [56].

## 3. CRISPR Screens in PCa

To date, several CRISPR screens have been reported in PCa. This section will present the results of the categorization of each type of screen (Table 1).

### 3.1. Discovery of Potential Target

The first CRISPR-KO screen was published back-to-back in early 2014 [17]. Then, Fei et al. reported the first paper regarding genome-wide CRISPR-KO screen in PCa cells in 2017 [57]. They carried out a viability-based screen with GeCKO v2 library in LNCaP cells and identified HNRLP as an essential gene required for PCa growth using their custom-developed MAGeCK and MAGeCK-VISPR algorithms [48,49]. HNRNPL directly regulates its RNA targets, including AR-encoding ones, through either linearly alternative splicing or back-spliced circRNA formation. Importantly, both HNRNPL and its RNA targets are aberrantly expressed in PCa, supporting their clinical relevance. They concluded that HNRLP is a potential therapeutic target in PCa. Similarly, CRISPR-KO screen using nuclear protein sgRNA sub-pool library uncovered that heterozygous deletion of 17p, which is frequently detected (up to 63%) in metastatic PCa cohort, confers a selective dependence on RBX1 [58]. The concurrent inhibition of RNAP2 and RBX1 suppresses the growth of CRPC in a synergistic manner, which potentiates the therapeutic efficacy of the RNAP2 inhibitor, α-amanitin-conjugated anti-EpCAM antibodies. Aquirre et al. performed a genome-scale loss-of-function genetic screen in 33 cancer cell lines including PCa [59]. Yoshiyama et al. followed up by analyzing data of their screens in PCa cell lines as obtained from a public resource of CRISPR-KO screens [60], Cancer Dependency Map (DepMaP) [61]. They identified histone demethylase JMJD1C (KDM3C) as an AR-negative context-specific vulnerability. In several PCa models, they demonstrated that JMJD1C depletion leads to specific growth suppression of AR-negative cells through activation of the tumor necrosis factor alpha (TNFα) network. Finally, they concluded that the identification of JMJD1C inhibition as a specific vulnerability in AR-negative PCa may provide an alternative drug target for PCa patients progressing on AR inhibitor therapy.

Das et al. conducted the first genome-scale CRISPRi screen in metastatic PCa models to identify genes required for cell survival [62]. They demonstrated that Kinesin Family Member 4A (KIF4A) and WD Repeat Domain 62 (WDR62) promote aggressive PCa phenotypes in vitro and in vivo, leading to nominating KIF4A and WDR62 as PCa driver genes, combined with their clinical data.

Marker selection screen, a CRISPR-based E3 ligase screening approach in DsRed/EGFP-PDK1 reporter HKT cells carried out by Jian et al. identified that Cullin3SPOP E3 ligase promotes PDK1 ubiquitination and subsequent degradation [63]. Of note, around 15% of the SPOP mutations have been identified in PCa setting [64,65,66]. They investigated using PCa cell lines where mechanistically, SPOP recognizes PDK1 in a CK1/GSK3β-mediated phosphorylation and degron dependent manner. Either loss-of-function mutations of SPOP or gain-of- function mutations of PDK1 in their binding region all attenuate SPOP recognizing and ubiquitinating PDK1, leading to elevated PDK1 protein abundance, AKT kinase activity, and benefit of tumor malignancies, indicating that the PDK1-AKT pathway will be a potential target for mutated SPOP- or PDK1-driven PCa.

**Table 1:** Results of CRISPR screens for prostate cancer.

Study	Screen Type	Library (Number of Genes)	Cell Line	Algorithm	Biomarkers	Results
Fei et al. (2017) [57]	Knockout	GeCKO v2 (19,050)	LNCaP	MAGeCK, MAGeCK-VISPR	HNRNPL	HNRNPL and its RNA clients as players in PCa growth and potential therapeutic targets.
Li et al. (2018) [58]	Knockout	Nuclear proteins sgRNA sub-pool library (3733)	DU145, 17p loss-DU145	edgeR	RBX1	Heterozygous deletion of 17p confers a selective dependence on RBX1.
Aquirre et al. (2016) [59] analyzed by Yoshiyama et al. (2021) [60]	Knockout	GeCKO v2 (19,050)	LNCaP, PC3	BAGEL	JMJD1C	JMJD1C depletion leads to specific growth suppression of AR-negative cells via activation of the TNFα network.
Das et al. (2021) [62]	Knockdown	Human CRISPRi v2 Top5 sgRNA library (18,905)	LNCaP, C4-2B	ScreenProcessing	KIF4A, WDR62	KIF4A and WDR62 drive aggressive prostate cancer phenotypes irrespective of AR-status.
Jiang et al. (2021) [63]	Knockout	E3 ubiquitin ligase contained CRISPR/Cas9 library (943)	EGFP-PDK1 reporter HEK293	Not shown	SPOP	PDK1 underwent SPOP-mediated ubiquitination and subsequent proteasome-dependent degradation, which suppresses AKT kinase activity and oncogenic functions.
Palit et al. (2019) [67]	Knockout	GeCKO library A (19,052)	LNCaP	MAGeCK	TLE3	Loss of TLE3 confers resistance to AR antagonists apalutamide and enzalutamide.
Palit et al. (2021) [68]	Knockout	NKI Human Kinome CRISPR pooled sgRNA library (578)	CWR-R1	MAGeCK	BRAF	BRAF contribute to resistance ton AR targeted therapy in PCa. BRAF mutated patients is candidate for AR inhibitors.
Lei et al. (2021) [69]	Knockout	kinome CRISPR library (507)	C4-2	MAGeCK	CDK12	CDK12 is a conservative vulnerability of PCa cells. The synergy of THZ531 and AR antagonists suggests a potential combination therapy for PCa.
Zimmermann et al. (2018) [70]	Knockout	TKOv1 (17,661)	Hela, RPE1-hTERT, SUM149PT	DrugZ, MAGeCK	RNASEH2	Mutations in all three genes encoding RNASEH2 sensitized cells to PARP inhibition.
Wang et al. (2019) [71]	Knockout	TKOv3 (18,053)	293A, HCT116, MCF10A	BAGEL	RNASEH2	RNASEH2 deficiency is synthetic lethal with ATR inhibition both in vitro and in vivo.
Chen et al. (2020) [72]	Activation	CRISPR/Cas9 Synergistic Activation Mediator (SAM) pooled library (23,430)	DU145, PC-3	Not shown	RAD9A	The activation of RAD9A contributed to in vitro resistance to metformin.
Chu et al. (2021) [73]	Knockout	GeCKO v2 library A (19,050)	M231-ADIR (ADI resistant MDA-MB)	Subread aligner, DESeq2.	TRME1/CCL2	TREM1/CCL2 activation, in addition to restored ASS1 expression, as a key pathway involved in full ADI-resistance in breast and prostate cancer models.

PCa: prostate cancer, AR: androgen receptor, ADI: arginine deiminase, PARP: poly (ADP-ribose) polymerase.

### 3.2. Discovery of Drug-Induced Synthetic Lethality Targets and Resistance Mechanisms

ADT is used to treat locally advanced and metastatic PCa, achieving remission in most patients. Although ADT offers near-certain remissions lasting 1–2 years in most patients, cancer cells become resistant with the emergence of CRPC [5]. AR signaling plays a pivotal role in CRPC, as evidenced by the effectiveness of AR-inducing drugs such as abiraterone and enzalutamide. Unfortunately, patients develop resistance to these drugs and invariably succumb to the disease [74,75,76].

Through a genome-wide CRISPR/Cas9 screen, Palit et al. identified a transducin-like enhancer of split 3 (TLE3) as a modulator of AR inhibitor sensitivity that, upon loss, confers resistance to enzalutamide in PCa LNCaP cells [67]. Interestingly, consistently with the binding of TLE3 and AR at the GR locus, TLE3 loss results in upregulation of glucocorticoid receptor (GR) expression. Their results provide novel insights into the regulation of the GR locus in the context of AR inhibition in PCa cells, implicating TLE3 as a regulator of GR-mediated AR inhibitor resistance. Using a similar approach, this group then set out to identify kinases whose inhibition could potentiate enzalutamide efficacy in PCa cells, with the aim to discover genes related to resistance and potential drug combinations which are able to overcome enzalutamide resistance [68]. They found that inhibition of BRAF, or downstream MAPK components MEK and ERK, enhanced enzalutamide sensitivity in PCa cells harboring a mutation in the activating kinase domain of the BRAF gene. These findings suggest therapeutic potential for co-inhibition of the MAPK and AR pathways in BRAF-mutated PCa.

Lei et al. performed a kinome-scale CRISPR/Cas9 screen and identified cyclin-dependent kinase 12 (CDK12) as essential for PCa cell viability [69]. THZ531, an inhibitor of CDK12, produced a marked anti-tumor effect. Mechanistically, THZ531 downregulated AR signaling and preferentially repressed CDK12 inhibition-sensitive transcripts (CDK12-ISTs). Furthermore, they revealed that THZ531 showed a remarkable synergistic effect with multiple AR antagonists.

Drugs targeting DDR pathways taking advantage of clinical synthetic lethality have already exhibited therapeutic benefit in several types of cancers [77,78]. Olaparib has also shown benefit in metastatic PCa in DDR-deficient patients, expanding the potential biomarkers of response beyond BRCA [9,10]. Other molecules and combinations of drugs such as ATR, ATM, CHK1 and DNA-PK, which aim to inhibit DDR, have also been well studied [79,80,81,82,83]. Zimmermann et al. performed a CRISPR-KO screen with PARP inhibitor olaparib treatment in broadly three different cell lines (Hela, RPE-hTERT and SUM149PT) and presented a high-confidence set of 73 genes, which, when mutated, cause increased sensitivity to PARP inhibitors [70]. Finally, they discovered that loss of ribonuclease H2 (RNASEH2) sensitized cells to PARP inhibition in a manner that impedes ribonucleotide excision repair in cells lacking ribonuclease H2, resulting in PARP trapping lesions that impair DNA replication and compromise genomic integrity. Notably, RNASEH2 has also identified loss of which causes synthetic lethality with ATR inhibitors through another CRISPR-KO screen [71]. Genome-wide KO screens performed with ATR inhibitor in several types of cancer cell lines (293A, HCT116 and MCF10A) demonstrated that RNASEH2 deficiency induces ATR inhibitor sensitivity both in vitro and in vivo. Although both studies performed CRISPR screen in non-PCa cancer cell lines, they demonstrated that loss of RNASEHEB, which is particularly frequent (up to 20% or more) in the PCa cohort, leads to synthetic lethality by PARP or ATR inhibition, indicating that RNASEH2B status is a potential determinant for treatment with both drugs in PCa patients.

Metformin, an oral biguanide drug used as a first-line treatment for type 2 diabetes, has attracted attention for its antiproliferative and anticancer effects on several solid tumors, including PCa [84,85]. However, conflicting results have been reported regarding the association between metformin use and the risk of developing PCa and survival [86]. Currently, additional effects of metformin in combination with drugs targeting the androgen receptor axis are being investigated in patients with CRPC and patients receiving salvage RT after RP. Overall, the relationship between metformin utilization and PCa is still controversial. Chen et al. provided new evidence to confirm the genetic determinants of metformin resistance in DU145 cells by conducting a genome-wide CRISPR/Cas9 activation screen using SAM pooled libraries [72]. The results showed that ECE1, ABCA12, BPY2, EEF1A1, RAD9A and NIPSNAP1 contribute to metformin resistance in PCa cells. They further demonstrated that RAD9A might be involved in the tumor immune microenvironment (TIME) modulation of metformin by upregulating regulatory T cells.

A pegylated arginine deiminase (ADI), ADI-PEG20, has been investigated in at least 20 Phase I/II clinical trials, including PCa, and has demonstrated an excellent safety profile [87,88,89]. Recent trials have shown medical benefits in combination with other agents in a number of cancers [90,91,92]. To identify novel factors conferring treatment resistance, Chu et al. conducted a genome-wide CRISPR-KO screen in ADI-resistant M231 cells treated with ADI and identified the TREM-CCL2 pathway as a critical factor in resistance through activation of the AKT/mTOR/STAT3/CCL2 pathway, combined with RNA-seq analysis [73]. They further validated the results also in PC3 PCa cell models. This study reveals a new pathway of ADI resistance and provides a new target to overcome ADI resistance in breast cancer and PCa.

## 4. Conclusions

Recent advances in genome sequencing have provided new insights into the molecular landscape of CRPC. The detection of DNA repair defects, such as mutations in the BRCA2, could guide the selection of patients to be treated with platinum chemotherapy [93,94] or poly (ADP-ribose) polymerase (PARP) inhibitors [9], while loss of mismatch repair genes and microsatellite instability have been reported as modulator of sensitivity to immunotherapy with checkpoint inhibitors [95]. Features of poor prognosis, such as the presence of RB1 deletion [96,97], might guide future treatment strategies.

Genome-wide CRISPR/Cas9 screen provides a robust and unbiased means for interrogating such genes, and a series of landmark reports since its introduction in 2014 have demonstrated that the technology yields high-quality functional hits [12]. This technology, in combination with other orthogonal methods (e.g., mass spectrometry) for studying protein function on a systems scale, can provide valuable functional insights that would take years to establish using conventional methods. This review finally presented several screens results to date regarding PCa. Since this field has some specific barriers, including the limited number of cell lines, it is critical to carefully understand the results of screening with regard to the clinical relevance to extrapolate to clinical setting. Even taking this into account, the studies presented here have the potential to be of clinical importance. Of note, some CRISPR screens in other cancer cell lines also provide novel targets for PCa [63,70,71], suggesting the importance of monitoring the results of screens in any type of cancer cells, which may lead to the discovery of biomarkers for CRPC therapy. In this regard, data portals such as DepMap provide assistance since there have been implementations of CRISPR screens in a multi-cancer lineage fashion in hundreds of cell lines including PCa. As mentioned above, their data analyses of other tumors may be useful for PCa, and vice versa. Further studies in PCa patients in the near future are warranted regarding the new targets from the screens presented in this paper.

Our understanding of the molecular features of CRPC through genomic research such as CRISPR screen will be applied to the clinic in the form of increased molecular testing for the use of agents and for clinical trial eligibility. Accordingly, CRPC patients will increasingly benefit from our understanding of the role of genomic alterations in the etiology of PCa and the potential for treating patients with specific mutations. Hopefully, this review will contribute to that.

## Abbreviations

PCa	Prostate cancer
ADT	androgen deprivation therapy
mCRPC	metastatic castration-resistant prostate cancer
AR	androgen receptor
CRISPR	clustered regularly interspaced short palindromic repeats
RNAi	RNA interference
WT	wild-type
dCas9	dead Cas9
CRISPRa	CRISPR activation
CRISPRi	CRISPR interference
CRPC	castration-resistant prostate cancer
sgRNA	single guide RNAs
MOI	multiplicity of infection
FACS	fluorescence-activated cell sorting
NGS	next-generation sequencing
MAGeCK	Model based Analysis of Genome-wide CRISPR/Cas9 Knockout
BAGEL	Bayesian Analysis of Gene EssentiaLity
caRpools	CRISPR AnalyzeR for Pooled Screens
PinAPL-Py	Platform-independent Analysis of Pooled Screens using Python
DepMaP	Cancer Dependency Map
TNFα	tumor necrosis factor alpha
KIF4A	Kinesin Family Member 4A
WDR62	WD Repeat Domain 62
TLE3	transducin-like enhancer of split 3
GR	glucocorticoid receptor
CDK12	cyclin-dependent kinase 12
CDK12-ISTs	CDK12 inhibition-sensitive transcripts
TIME	tumor immune microenvironment
ADI	arginine deiminase
PARP	poly(ADP-ribose) polymerase
